# Spatial distribution of IL4 controls iNKT cell-DC crosstalk in tumors

**DOI:** 10.1038/s41423-019-0243-z

**Published:** 2019-06-03

**Authors:** Lu Wang, Zhilan Liu, Lili Wang, Qielan Wu, Xiang Li, Di Xie, Huimin Zhang, Yongdeng Zhang, Lusheng Gu, Yanhong Xue, Ting Yue, Gang Liu, Wei Ji, Haiming Wei, Tao Xu, Li Bai

**Affiliations:** 1 Division of Life Sciences and Medicine, Department of Oncology of The First Affiliated Hospital, The CAS Key Laboratory of Innate Immunity and Chronic Disease, University of Science and Technology of China, 230027 Hefei, China; 2 National Key Laboratory of Biomacromolecules, Institute of Biophysics, Chinese Academy of Sciences, 100101 Beijing, China; 3 School of Life Sciences, University of Science and Technology of China, 230027 Hefei, China; 4 National Synchrotron Radiation Laboratory, University of Science and Technology of China, 230027 Hefei, China

**Keywords:** Spatial distribution, Polarization, IL4, iNKT, DC, Crosstalk, Tumor, Cytokines, Signal transduction, Membrane trafficking, Tumour immunology

## Abstract

The spatiotemporal distribution of cytokines orchestrates immune responses in vivo, yet the underlying mechanisms remain to be explored. We showed here that the spatial distribution of interleukin-4 (IL4) in invariant natural killer T (iNKT) cells regulated crosstalk between iNKT cells and dendritic cells (DCs) and controlled iNKT cell-mediated T-helper type 1 (Th1) responses. The persistent polarization of IL4 induced by strong lipid antigens, that is, α-galactosylceramide (αGC), caused IL4 accumulation at the immunological synapse (IS), which promoted the activation of the IL4R-STAT6 (signal transducer and activator of transcription 6) pathway and production of IL12 in DCs, which enhanced interferon-γ (IFNγ) production in iNKT cells. Conversely, the nonpolarized secretion of IL4 induced by Th2 lipid antigens with a short or unsaturated chain was incapable of enhancing this iNKT cell-DC crosstalk and thus shifted the immune response to a Th2-type response. The nonpolarized secretion of IL4 in response to Th2 lipid antigens was caused by the degradation of Cdc42 in iNKT cells. Moreover, reduced Cdc42 expression was observed in tumor-infiltrating iNKT cells, which impaired IL4 polarization and disturbed iNKT cell-DC crosstalk in tumors.

## Introduction

The release of cytokines is highly regulated in both the spatial and temporal dimensions to orchestrate immune responses. Cascades of cytokines promote crosstalk between distinct immune cells and mount appropriate immune responses in the proper location. Different cytokine secretory routes are utilized by immune cells. It has been shown that interferon-γ (IFNγ) and interleukin-2 (IL2) in T-helper type 1 (Th1) cells,^1^ IL10 in Th2 cells,^1^ lytic granules in natural killer (NK) cells,^2,3^ IL12 in dendritic cells (DCs),^4,5^ and lysosomes in B cells^6^ are directed to immunological synapses (ISs). This polarized secretion efficiently delivers their cargo to the IS, which enables specific targeting and promotes crosstalk between two cells forming an IS.^1,7^ On the other hand, multidirectional secretion of tumor necrosis factor-α (TNFα) and IL4 is observed in Th1 and Th2 cells, respectively, which causes the dispersal of cytokines and regulates only the cells able to respond to low concentrations of cytokines.^1^ The factors controlling secretory routes and functional significance of polarized secretion and multidirectional secretion remain unclear. Spatial alteration of cytokine secretory sites would predictably shift immune responses in vivo by modulating the functions of distinct responder cells.

As important immune regulators, invariant NK T (iNKT) cells release both Th1 and Th2 cytokines (IFNγ and IL4, respectively)^8^ and bridge the innate and adaptive immune responses upon recognition of lipid antigens presented by CD1d molecules.^9,10^ Altered iNKT cell functions have been reported during the progression of diseases.^11,12^ Under certain conditions, the activation of iNKT cells will cause either Th1- or Th2-biased responses that are critically related to disease progression and intervention. For example, IFNγ production by iNKT cells promotes the clearance of pathogens^13^ and inhibits tumor growth.^14,15^ On the other hand, iNKT cells shift their functions toward Th2 responses by producing large amounts of IL4 but small amounts of IFNγ,^16,17^ and this change favors tumor growth,^18^ but inhibits autoimmune diseases.^17,19,20^ The mechanisms underlying biased iNKT cell functions are not fully understood. Whether cytokine secretory routes regulate iNKT cell-mediated immune responses remains unclear.

Here, we demonstrate that the spatial distribution of IL4 shapes iNKT cell functions. The secretory routes of IL4 in iNKT cells were controlled by the type of lipid antigen. Unlike the most potent lipid antigen α-galactosylceramide (αGC), several lipid antigen variants with a short or unsaturated chain induce Th2-biased immune responses.^16^ αGC maintained the reorientation of the microtubule-organizing center (MTOC) toward the IS and thus stabilized IL4 polarization. Polarized secretion concentrated IL4 at the IS, which promoted the activation of signal transducer and activator of transcription 6 (STAT6) and production of IL12 in DCs and therefore favored IFNγ production in iNKT cells. Conversely, multidirectional secretion of IL4 induced by Th2 lipid antigens failed to promote this iNKT cell-DC crosstalk. Taxol stabilized the MTOC and prolonged Th2 lipid antigen-induced IL4 polarization to significantly augment Th1 responses in vitro. Moreover, we showed that multidirectional secretion in response to Th2 lipid antigens resulted from a reduction in Cdc42 expression. The restoration of active Cdc42 successfully prolonged Th2 lipid antigen-induced polarization of the MTOC and IL4 production. Additionally, the reduction in Cdc42 expression was detected in intratumoral iNKT cells and interfered with IL4 polarization and crosstalk between iNKT cells and DCs in tumors.

## Results

### αGC, not Th2 lipid antigen variants, induces activation of the IL4R-STAT6 pathway in DCs

**Fig. 1:**
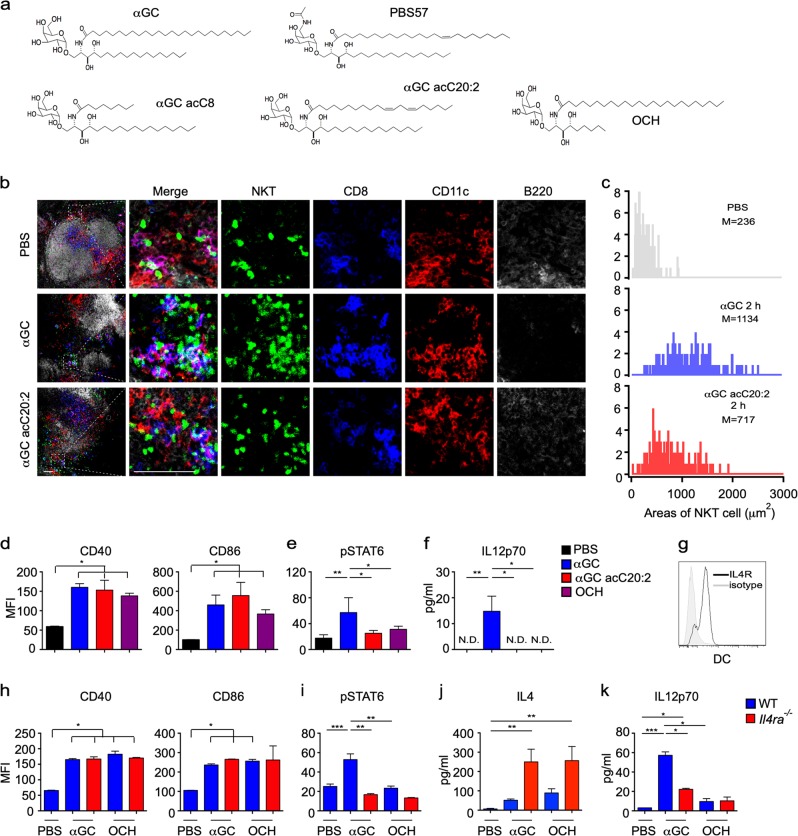
α-Galactosylceramide (αGC), not T-helper type 2 (Th2) lipid antigens, activates the IL4 receptor-signal transducer and activator of transcription 6 (IL4R-STAT6) pathway in dendritic cells (DCs.) **a** Structures of lipid antigen variants. **b** In vivo colocalization of invariant natural killer T (iNKT) cells and DCs in the spleen of *Vα14* Tg. *Cxcr6*^*gfp/+*^ mice 2 h after injecting αGC or αGC acC20:2 (2 μg per mouse, intraperitoneally (i.p.)). Blue, CD8; red, CD11c; gray, B220; and green, iNKT. Scale bars, 50 μm. Data are representative of three independent experiments. **c** Distribution of the total area occupied by iNKT cells in each 100 μm × 100 μm DC zone (*n* ≥ 160 zones per group). Data are pooled from three independent experiments. M indicates the mean area value. **d**–**f** Expression of CD40 and CD86 (**d**) and phosphorylation of STAT6 (**e**) in splenic CD11c^+^ DCs and production of IL12p70 in the serum (**f**) of wild-type (WT) mice 8 h after receiving the indicated lipid antigens. Data are presented as the mean ± SEM of five to six mice per group. **g** Expression of IL4Rα in splenic CD11c^+^ DCs. Data are representative of five independent experiments. **h**–**k** Expression of CD40 and CD86 (**h**) and phosphorylation of STAT6 (**i**) in splenic CD11c^+^ DCs and serum production of IL4 (**j**) and IL12p70 (**k**) in WT or *Il4a*^*−/−*^ mice 8 h after receiving the indicated lipid antigens. Data are presented as the mean ± SEM of five to six mice per group. Statistical analysis was performed using one-way analysis of variance (ANOVA) with the Tukey’s post test. *P < 0.05; **P < 0.01; and ***P < 0.001

αGC (Fig. 1a) is a strong lipid antigen that activates iNKT cells and induces both IL4 and IFNγ production in vivo, whereas OCH, αGC acC8, and αGC acC20:2 (Fig. 1a), which have a truncated or unsaturated acyl chain or sphingosine chain, induce mainly IL4 production in vivo and are therefore regarded as Th2 lipid antigens.^21,22^ Due to reciprocal interactions between iNKT cells and antigen-presenting cells (APCs), the type of APC shapes the functions of the iNKT cells.^11,22–24^ We investigated the interactions between iNKT cells and APCs in vivo. Two hours after injecting αGC, iNKT cells formed clusters exclusively with DCs, especially CD8^+^ DCs (Fig. 1b). On the other hand, Th2 lipid antigens are efficiently presented by distinct cell types.^22^ Clusters of iNKT cells around CD8^+^ DCs were also detected 2 h after injecting the Th2 lipid antigen αGC acC20:2 (Fig. 1b). Due to the formation of the clusters, it was difficult to count the absolute numbers of iNKT cells. Therefore, we measured the total area occupied by the iNKT cells in a 100 µm × 100 μm square where CD8^+^ DCs were enriched. The area occupied by the iNKT cells in DC zones was increased 2 h after injecting αGC. An increased area occupied by iNKT cells in DC zones was also detected after injecting αGC acC20:2 (Fig. 1c). These results indicate that both αGC and the Th2 lipid antigen αGC acC20:2 are able to induce interactions between DCs and iNKT cells. Consistently, similar activation of DCs by αGC and by Th2 lipid antigens, including αGC acC20:2 and OCH, was confirmed, as indicated by comparable upregulation of CD40 and CD86 expression (Fig. 1d). However, αGC and Th2 lipid antigens differed in their capability to induce the activation of STAT6 in DCs in vivo. Only αGC, not Th2 lipid antigens, caused the phosphorylation of STAT6 (Fig. 1e). In agreement with previous findings that indicated that the activation of the STAT6 pathway promotes IL12 production in DCs,^25^ we detected IL12 production in mice injected with αGC, but not in those injected with Th2 lipid antigens (Fig. 1f). The activation of STAT6 is induced by the Th2 cytokines IL4 and IL13.^25^ High expression of IL4 receptor α (IL4Rα) was detected in DCs (Fig. 1g). When splenic DCs were stimulated with lipopolysaccharide (LPS) in vitro, IL4 significantly increased the phosphorylation of STAT6 and production of IL12 in a dose-dependent manner (Supplementary Fig. S1A, B), but showed no effect in the expression of costimulatory molecules, including CD40, CD80, and CD86 (Supplementary Fig. S1C). Since iNKT cells produce a large amount of IL4 after activation, it is possible that the activation of STAT6 and production of IL12 in DCs induced by αGC are promoted by IL4 from iNKT cells. Next, we measured the activation of STAT6 in *Il4ra*^*−/−*^ mice. IL4R deficiency showed no influence on lipid antigen-induced upregulation of CD40 and CD86 expression in splenic DCs in vivo (Fig. 1h). However, in these *Il4ra*^*−/−*^ mice, αGC failed to increase the phosphorylation of STAT6 in the splenic DCs (Fig. 1i). These results confirmed that αGC induced STAT6 activation through IL4R signaling. On the other hand, more IL4 was detected in the serum of these IL4R-deficient mice (Fig. 1j), excluding the possibility that the inhibition of STAT6 activation was due to insufficient IL4 production. In agreement with the role of the IL4-STAT6 pathway in promoting IL12 production, αGC-induced IL12 production was significantly inhibited in the *Il4ra*^*−/−*^ mice (Fig. 1k). Together, our results demonstrate that lipid antigen variants differ in their capability to activate the IL4R-STAT6 pathway in DCs, which promotes the production of IL12.

### IL4 signaling promotes DC-iNKT cell crosstalk and iNKT cell-mediated Th1 responses

**Fig. 2:**
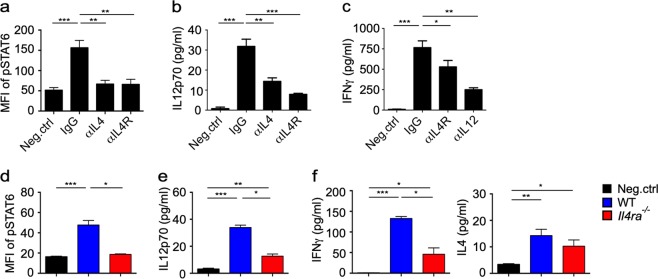
Interleukin-4 (IL4) promotes invariant natural killer T (iNKT) cell-dendritic cell (DC) crosstalk and T-helper type 1 (Th1) responses. **a**–**c** Phosphorylated signal transducer and activator of transcription 6 (STAT6) in CD11c^+^ DCs (**a**) and IL12p70 (**b**) and IFNγ levels (**c**) in supernatant. iNKT cells were activated for 8 h by DCs pulsed with αGC (1 μg/ml) with or without immunoglobulin G (IgG) 2b (isotype control), an anti-IL4 antibody, an anti-IL4R antibody, and an anti-IL12 antibody. Data are presented as the mean ± SEM of more than nine biological replicates. **d**–**f** Phosphorylated STAT6 in carboxyfluorescein succinimidyl ester-positive (CFSE^+^) BMDCs in the spleen (**d**) and levels of IL12p70 (**e**), IFNγ, and IL4 in the serum (**f**) of WT recipient mice 8 h after intravenously (i.v.) injecting α-galactosylceramide (αGC)-pulsed WT or *Il4ra*^*−/−*^ CFSE^+^ BMDCs. Data are presented as the mean ± SEM of five mice per group. Statistical analysis was performed using one-way analysis of variance (ANOVA) with the Tukey’s post test. *P < 0.05; **P < 0.01; and ***P < 0.001

In agreement with the in vivo studies, when αGC-pulsed DCs were cocultured with iNKT cells, an anti-IL4R antibody and anti-IL4 antibody significantly blocked the activation of STAT6 (Fig. 2a) and reduced the production of IL12 (Fig. 2b) in DCs in vitro. IL12 has previously been shown to promote the Th1 response in iNKT cells.^26^ Here, we found that the reduction in IL12 expression further dampened IFNγ production by the iNKT cells in this coculture system, as indicated by reduced IFNγ production in the presence of an anti-IL4R-blocking antibody or anti-IL12 antibody (Fig. 2c). To exclude the direct influence of IL4 signaling on iNKT cells, wild-type (WT) bone marrow-derived dendritic cells (BMDCs) and *Il4ra*^*−/−*^ BMDCs were generated and transferred into WT recipient mice after the cells were loaded with αGC and carboxyfluorescein succinimidyl ester (CFSE). In comparison to the αGC-loaded WT BMDCs, the αGC-loaded *Il4ra*^*−/−*^ BMDCs exhibited less activation of STAT6 (Fig. 2d) and caused less production of IL12 (Fig. 2e) and IFNγ (Fig. 2f) in the serum. These results demonstrated that IL4R signaling in DCs was important for DC-iNKT cell crosstalk and Th1 responses mediated by iNKT cells.

### Distinct secretory sites for IL4 are detected in iNKT cells in response to different lipid antigen variants

**Fig. 3:**
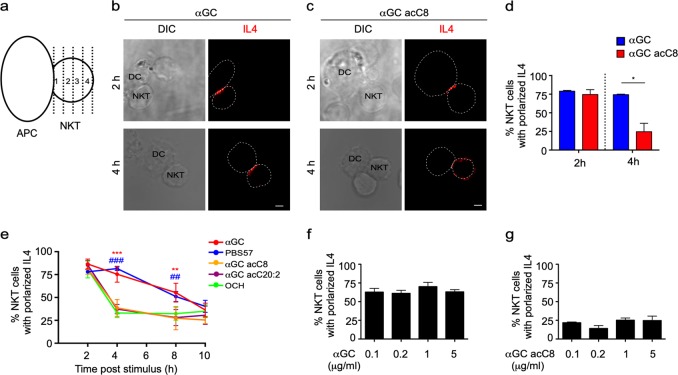
α-Galactosylceramide (αGC), not T-helper type 2 (Th2) lipid antigens, maintains the polarized secretion of IL4 in invariant natural killer T (iNKT) cells. **a** Method to quantify the polarized secretion of IL4. IL4 in area 1 is defined as polarized secretion. **b**, **c** Secretory sites of IL4 in iNKT cells activated by distinct antigen variant-pulsed splenic dendritic cells (DCs) at the indicated time points. Dotted lines indicate cell boundaries. Scale bars, 2 μm. Data are representative of three independent experiments and more than 70 cells per group. **d** Frequency of iNKT cells with polarized IL4 secretion. Data are presented as the mean ± SEM of three independent experiments. **e** Frequency of iNKT cells with polarized IL4 secretion after activation by distinct antigen variant-pulsed RBL.CD1d cells at the indicated time points. Data are presented as the mean ± SEM of three independent experiments. * indicates a significant difference between αGC and Th2 lipids, and ^#^ indicates a significant difference between PBS57 and Th2 lipids. **f**, **g** Frequency of polarized IL4 secretion at the IS induced by the indicated concentrations of αGC (**f**) and αGC acC8 (**g**) 4 h after activation. Data are presented as the mean ± SEM of three independent experiments. Statistical analysis was performed using Student’s *t* -test, two-way analysis of variance (ANOVA) or one-way ANOVA with the Tukey’s post test. *P < 0.05; ** or ^##^*P* < 0.01; and *** or ^###^*P* < 0.001

Th2 lipids failed to activate the IL4R-STAT6 pathway in DCs (Fig. 1e, i), and this failure was not due to insufficient IL4 production (Fig. 1j).^22^ In addition to the amounts of cytokines, the spatial distributions of cytokines also influence immune responses. To investigate cytokine secretory directions, we labeled the cell surface with a cytokine-specific capture reagent, which captures the cytokine while it is released. Due to the higher local concentration, the cytokine will be more efficiently captured at secretory sites than at other sites (Supplementary Fig. S2A). Thus, a detection antibody specific for the cytokine could be used to indicate the sites of secretion. No signal was detected in cells without cytokine secretion or in the absence of the capture reagent (Supplementary Fig. S2B), which confirmed the specificity of the detection antibody and excluded the possibility of capturing the cytokine by endogenous receptors. The capture reagent labels the cell surface by binding to CD45.^27^ Although CD45 was equally distributed on the cell surface, the cytokine was detected at immune synapses between iNKT cells and APCs (Supplementary Fig. S2B), excluding the possibility that the location of the detection antibody indicated the distribution of CD45 rather than that of the cytokine secretory sites. iNKT cells were cocultured with αGC- or αGC acC8-pulsed DCs, and cytokine secretory sites were detected at the indicated time points with the approach described above. We divided the iNKT cells into four parts with equal interval lines parallel to the synaptic interface, and IL4 release within the first part was defined as polarized secretion (Fig. 3a). Polarized secretion of IL4 at the IS was induced by both αGC and αGC acC8 after activating cells for 2 h. However, αGC but not αGC acC8 maintained the polarization of IL4 after 4 h of activation (Fig. 3b, c). The percentage of iNKT cells that released IL4 at the IS decreased from 85% to 27% in response to αGC acC8 (Fig. 3d). When artificial APCs, RBL.CD1d cells, were used instead of DCs to activate iNKT cells, similar results were observed (Fig. 3e). PBS57, another strong lipid antigen that induces a large IFNγ response in vivo,^28^ maintained the polarization of IL4 as efficiently as αGC. Even after 8 h of activation, more than 50% of the iNKT cells activated by αGC or PBS57 released IL4 in a polarized manner (Fig. 3e). On the other hand, other Th2 lipids, such as αGC acC20:2 and OCH, induced transient polarization of IL4, but multidirectional secretion of IL4 was predominantly detected after activating the iNKT cells for 4 h (Fig. 3e). To exclude the influence of antigen quantity on cytokine secretory routes, APCs were loaded with different concentrations of αGC or αGC acC8. Our results demonstrated that the type of lipid variants, rather than the amount of lipid, controlled the secretory routes in iNKT cells (Fig. 3f, g).

### Different secretory routes activated in response to lipid antigen variants are controlled by the distribution of the MTOC

**Fig. 4:**
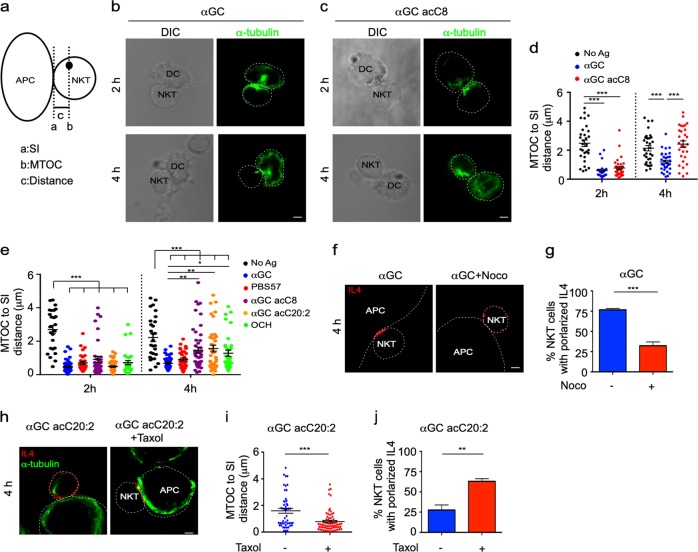
Microtubule-organizing center (MTOC) controls polarized secretion of interleukin-4 (IL4). **a** Method to quantify the distance from the MTOC to the synaptic interface (SI). **b**, **c** Polarization of the MTOC in invariant natural killer T (iNKT) cells activated by distinct antigen variant-pulsed splenic dendritic cells (DCs) at the indicated time points. Scale bars, 2 μm. Data are representative of three independent experiments and more than 60 cells per group. **d** Distance from the MTOC to the SI in the iNKT cells described in **b**, **c**. Data are presented as the mean ± SEM of more than 40 cells per group. **e** Distance from the MTOC to the SI in iNKT cells activated by distinct antigen variant-pulsed RBL.CD1d cells at the indicated time points. Data are presented as the mean ± SEM of more than 60 cells per group. **f**, **g** Influences of nocodazole (33 μM) on IL4 secretory sites (**f**) and the frequency of iNKT cells with polarized secretion (**g**) after activation by α-galactosylceramide (αGC)-pulsed RBL.CD1d cells for 4 h. Scale bars, 2 μm. Data are representative of three independent experiments (**f**) or are presented as the mean ± SEM of three independent experiments (**g**). **h**–**j** Influences of taxol (100 nM) on the distance from the MTOC to the SI (**h**, **i**) and on IL4 polarization (**h**, **j**) in iNKT cells activated by αGC acC20:2-pulsed RBL.CD1d cells for 4 h. Scale bars, 2 μm. Data are representative of three independent experiments (**h**), are presented as the mean ± SEM of (**j**) three independent experiments or are presented as the mean ± SEM of more than 60 cells per group (**i**). Dotted lines indicate cell boundaries. Statistical analysis was performed using one-way analysis of variance (ANOVA) with the Tukey’s post test or using Student’s *t* test. *P < 0.05; **P < 0.01; and ***P < 0.001

Reorientation of the MTOC toward an IS has been reported to regulate the polarization of vesicles.^29–32^ Next, we investigated the polarization of the MTOC in iNKT cells in response to distinct lipid antigen variants. Polarization of the MTOC reduces the distance from the MTOC to the synaptic interface.^33^ After activating iNKT cells for 2 h, lipid antigens significantly shortened the distance from the MTOC to the synaptic interface, indicating the polarization of the MTOC toward the synaptic interface (Fig. 4a–d). At this time point, the distances from the MTOC to the interface were similar between iNKT cells activated by αGC and those activated by the Th2 lipid antigen αGC acC8 (Fig. 4b–d). These results were in agreement with the polarization of IL4 observed at the early time point (Fig. 3b–d). However, after 4 h of activation, increased distances from the MTOC to the synaptic interface were observed in the iNKT cells activated by αGC acC8 compared to the cells activated by αGC (Fig. 4b–d). The increased distances indicated the redistribution of the MTOC away from the synapse, which was consistent with the multidirectional secretion of IL4 in iNKT cells activated by αGC acC8 (Fig. 3b–d). Moreover, after activating iNKT cells for 4 h, αGC acC20:2 and OCH, but not PBS57, caused a longer distance from the MTOC to the synaptic interface than did αGC (Fig. 4e). These results demonstrated that Th2 lipids were incapable of maintaining the polarization of the MTOC. To investigate whether the MTOC controlled the polarization of IL4, nocodazole, which interferes with the polymerization of microtubules, was added into the culture medium after activating iNKT cells to inhibit αGC-induced MTOC polarization. As a consequence, the percentage of cells with polarized secretion was significantly reduced from 75 to 25% (Fig. 4f, g). In contrast, when taxol was used to stabilize the MTOC after activating iNKT cells with αGC acC20:2, the polarization of the MTOC and IL4 was maintained (Fig. 4h–j). These inhibitors were added 2 h after T cell receptor (TCR) engagement to exclude their influence on cell activation. Furthermore, when fixed antigen-pulsed APCs were used to activate iNKT cells, similar results were detected, which excluded an effect of the inhibitors on the APCs (Supplementary Fig. S3). Overall, these results demonstrate that distinct secretory routes in response to lipid variants are controlled by the position of the MTOC in iNKT cells.

### Polarization of IL4 at the IS promotes the activation of STAT6 in DCs and iNKT cell-mediated Th1 responses

**Fig. 5:**
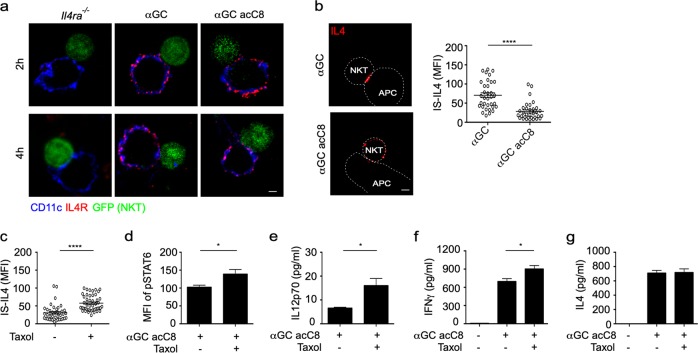
Taxol prolongs interleukin-4 (IL4) polarization in invariant natural killer T (iNKT) cells and promotes α-galactosylceramide (αGC) acC8-induced T-helper type 1 (Th1) responses. **a** Distribution of IL4R (red) on the surface of CD11c^+^ (blue) dendritic cells (DCs). iNKT (GFP^+^) cells were activated by distinct antigen variant-pulsed splenic DCs for 4 h. Scale bars, 2 μm. Data are representative of two independent experiments and more than 25 cells per group. **b** Accumulation of IL4 at the immunological synapse (IS) in response to αGC-pulsed or αGC acC8-pulsed splenic DCs. Dotted lines indicate cell boundaries. Scale bars, 2 μm. Data are representative of three independent experiments (left) or are presented as the mean ± SEM of more than 35 cells per group (right). **c** Influence of taxol (100 nM) on the Th2 lipid-induced accumulation of IL4 at the IS, as shown in Fig. 4h. **d**–**g** Influences of taxol (100 nM) on the phosphorylation of signal transducer and activator of transcription 6 (STAT6) in CD11c^+^ DCs (**d**) and on the production of IL12p70 (**e**), interferon-γ (IFNγ) (**f**), and IL4 (**g**) in the supernatant. iNKT cells were activated by αGC acC8-pulsed (1 μg/ml) DCs for 8 h. Data are presented as the mean ± SEM of three independent experiments. Statistical analysis was performed using the Mann–Whitney *U* test. *P < 0.05 and ****P < 0.0001

Although the secretory sites of IL4 in iNKT cells were controlled by lipid antigen variants, the distribution of IL4R on the DC surface was not influenced by these lipids (Fig. 5a). Polarized secretion concentrated IL4 at the IS, as indicated by αGC inducing a higher amount of IL4 at the IS than αGC acC8 (Fig. 5b). Next, we investigated whether the polarization of IL4 promoted crosstalk between DCs and iNKT cells. We forced polarization of IL4 in αGC acC8-activated iNKT cells by adding taxol after cell activation (Fig. 4h, j), and taxol significantly increased the accumulation of IL4 at synapses (Fig. 5c). As a result, taxol significantly increased STAT6 phosphorylation (Fig. 5d) and IL12 production (Fig. 5e) in αGC acC8-pulsed DCs. Notably, these results were not due to the direct influence of taxol on the DCs since taxol did not promote the activation of STAT6 and production of IL12 in DCs activated by LPS plus IL4 (Supplementary Fig. S4). Moreover, taxol augmented IFNγ production by iNKT cells (Fig. 5f). The production of IL4 was not changed by taxol (Fig. 5g), which further confirmed that taxol influenced iNKT cell-DC crosstalk by modulating the spatial distribution rather than the amount of IL4. Taken together, our results demonstrate that the polarization of IL4 at the IS favors crosstalk between iNKT cells and DCs and iNKT cell-mediated Th1 responses.

### Th2 lipid antigens disturb the polarization of the MTOC and IL4 by reducing the Cdc42 protein level in iNKT cells

**Fig. 6:**
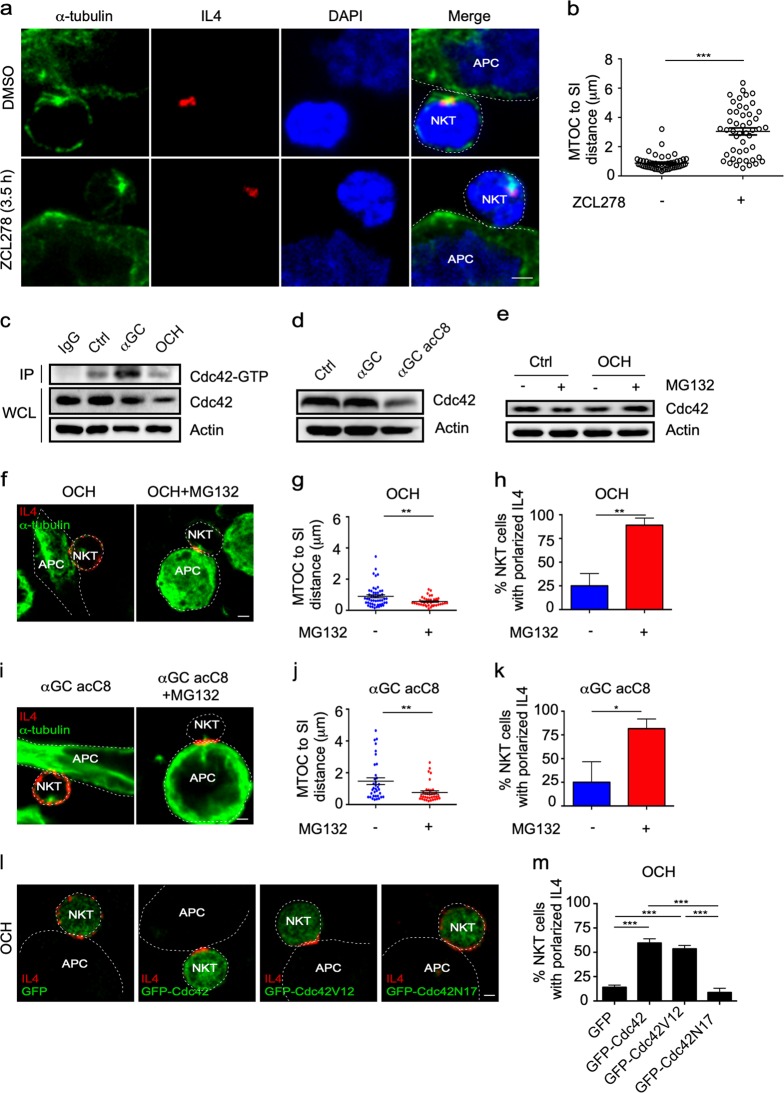
T-helper type 1 (Th2) lipid antigens disturb the polarization of the microtubule-organizing center (MTOC) by reducing Cdc42 expression in invariant natural killer T (iNKT) cells. **a**, **b** Influences of ZCL278 (100 μM) on MTOC polarization, intracellular interleukin-4 (IL4) polarization (**a**), and the distance from the MTOC to the synaptic interface (SI) (**b**) in iNKT cells activated by α-galactosylceramide (αGC)-pulsed RBL.CD1d cells for 4 h. ZCL278 was added to the culture medium in the last 30 min. Scale bars, 2 μm. Data are representative of three independent experiments (**a**) or are presented as the mean ± SEM of more than 45 cells per group (**b**). **c**, **d** Cdc42-GTP and total Cdc42 levels in iNKT cells activated by the indicated lipid antigen-pulsed RBL.CD1d cells for 4 h. Data are representative of three independent experiments. **e** Influence of MG132 (5 μM) on the Cdc42 level in iNKT cells activated by OCH-pulsed RBL.CD1d cells. Data are representative of three independent experiments. **f**–**k** Influences of MG132 (5 μM) on the polarization of the MTOC and IL4 (**f**, **i**), the distance from the MTOC to the SI (**g**, **j**), and the frequency of iNKT cells with polarized secretion (**h**, **k**) after activation by the indicated antigen-pulsed RBL.CD1d cells for 4 h. Scale bars, 2 μm. Data are representative of three independent experiments (**f**, **i**), are presented as the mean ± SEM of three independent experiments (**h**, **k**), or are presented as the mean ± SEM of more than 50 cells per group (**g**, **j**). **l**, **m** IL4 secretory sites (**l**) and the frequencies of iNKT cells overexpressing EGFP-Cdc42, EGFP-Cdc42V12, or EGFP-Cdc42N17 with polarized secretion (**m**) after activation by OCH-pulsed RBL.CD1d cells for 4 h. Data are representative of three independent experiments (**l**) or are presented as the mean ± SEM of (**m**) three independent experiments. Scale bars, 2 μm. Dotted lines indicate cell boundaries. Statistical analysis was performed using Student’s *t* test, the Mann–Whitney *U* test or one-way analysis of variance (ANOVA) with the Tukey’s post test. *P < 0.05; **P < 0.01; and ***P < 0.001

DGK and Cdc42 have been previously shown to regulate MTOC polarization in several cell types.^34–36^ In our studies, an inhibitor of DGK, DGK II, only inhibited the reorientation of the MTOC toward the IS if it was added before iNKT cells were activated by αGC-pulsed RBL.CD1d cells (Supplementary Fig. S5A, B). After activation, DGK II showed no influence on the polarization of the MTOC and intracellular IL4 in iNKT cells (Supplementary Fig. S5A, B). Therefore, DGK controlled the initiation of MTOC polarization, but was dispensable for maintaining polarization. On the other hand, ZCL278, an inhibitor of Cdc42, dramatically inhibited the polarization of the MTOC and intracellular IL4 and increased the distance from the MTOC to the synaptic interface, even when added to iNKT cells 3.5h after cell activation (Fig. 6a, b). These results indicated a critical role for Cdc42 in maintaining the polarized distribution of the MTOC at the IS. In agreement with the inability of OCH to maintain MTOC polarization, a lower amount of GTP-bound Cdc42 was detected in OCH-activated cells than in αGC-activated iNKT cells (Fig. 6c). Importantly, the total Cdc42 protein level was significantly reduced in the OCH-activated iNKT cells but not in the αGC-activated cells (Fig. 6c), which explained the lower activity of Cdc42 in response to OCH. A similar reduction in the Cdc42 protein level was observed in αGC acC8-activated iNKT cells (Fig. 6d). To test whether the reduction in the Cdc42 level was due to protein degradation, we used GM132 to inhibit proteasome function. MG132 significantly restored the Cdc42 protein level in OCH-activated iNKT cells (Fig. 6e). Moreover, MG132 successfully prolonged the polarization of the MTOC and IL4 in cells activated by Th2 lipids, including OCH and αGC acC8 (Fig. 6f–k). To further confirm the influence of the Cdc42 protein level on cytokine secretory routes in iNKT cells, we restored the Cdc42 protein level in cells activated with OCH by overexpressing Cdc42. The cells transfected with EGFP-Cdc42 or the constitutively active mutant EGFP-Cdc42V12 but not those transfected with EGFP maintained the polarization of the MTOC and IL4 in response to OCH (Fig. 6l, m). These results proved that recovery of the Cdc42 protein level was able to maintain polarized secretion in response to a Th2 lipid antigen. Notably, the overexpression of the dominant inactive mutant EGFP-Cdc42N17 failed to do so (Fig. 6l, m). Therefore, the activity of Cdc42 was required. Due to the role of Cdc42 in IS formation,^35^ overexpressing EGFP-Cdc42N17 impaired the activation of iNKT cells and thereby reduced the number of IL4-expressing iNKT cells (data not shown). Taken together, the results show that lipid antigen variants regulate the secretory routes of IL4 by modulating the protein level of Cdc42.

### Reduced Cdc42 expression in intratumoral iNKT cells is associated with impaired IL4 polarization and disturbed iNKT cell-DC crosstalk in tumors

**Fig. 7:**
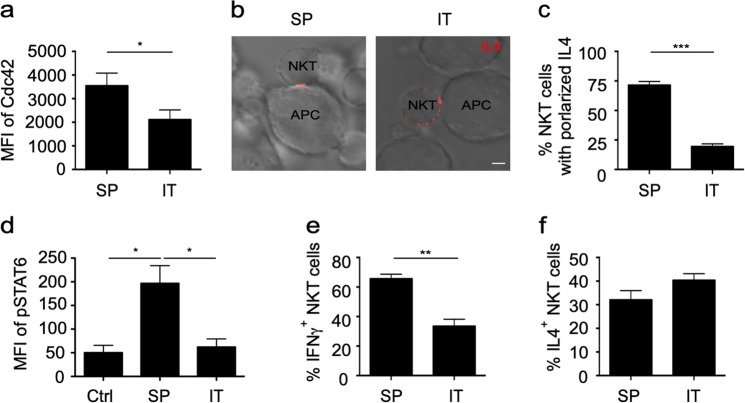
Impaired interleukin-4 (IL4) polarization and invariant natural killer T (iNKT) cell-dendritic cell (DC) crosstalk in tumors. **a** Expression of Cdc42 in intratumoral (IT) and splenic (SP) iNKT cells from MC38 tumor-bearing mice. Data are presented as the mean ± SEM of more than nine mice per group. **b**, **c** IL4 secretory sites (**b**) and the frequency of (**c**) intratumoral and splenic iNKT cells with polarized secretion after activation by αGC-pulsed RBL.CD1d for 4 h. Scale bars, 2 μm. Data are representative of three independent experiments (more than 60 cells per group, **b**) or are presented as the mean ± SEM of three independent experiments (**c**). **d–f** Phosphorylated signal transducer and activator of transcription 6 (STAT6) in splenic DCs and intratumoral MHC II^+^ CD24^+^ F4/80^−^ CD11c^+^ DCs (**d**) and the percentages of IFNγ^+^ (**e**) and IL4^+^ (**f**) iNKT cells in the tumor and spleen of MC38 tumor-bearing mice 8 h after injection with αGC. Data are presented as the mean ± SEM of more than nine mice. Statistical analysis was performed using the Mann–Whitney *U* test or Student’s *t* test. *P < 0.05; **P < 0.01; and ***P < 0.001

iNKT cell-mediated Th1 responses play important roles in tumor clearance.^37^ Reduced Cdc42 expression was detected in intratumoral iNKT cells compared to splenic iNKT cells from MC38 tumor-bearing mice (Fig. 7a). Consistently, unlike splenic iNKT cells, intratumoral iNKT cells failed to maintain the polarization of IL4 when they were activated by αGC-pulsed RBL.CD1d cells (Fig. 7b, c). Moreover, when tumor-bearing mice were injected with αGC, intratumoral DCs and iNKT cells showed less STAT6 phosphorylation (Fig. 7d) and IFNγ (Fig. 7e) production, respectively, than cells from the spleen, although similar production of IL4 was detected for the intratumoral iNKT cells and splenic iNKT cells (Fig. 7f). These results were in agreement with the important roles of IL4 polarization in activating STAT6 in DCs and promoting iNKT cell-DC crosstalk. Together, our results suggest that the reduction in Cdc42 expression in tumor-infiltrating iNKT cells disturbs IL4 polarization and interferes with iNKT cells-DC crosstalk, which contributes to the impaired antitumor Th1 responses mediated by iNKT cells.

## Discussion

IL12 plays an important role in promoting IFNγ production in iNKT cells and NK cells.^26,38,39^ Differences in IL12 production explain the biased cytokine profiles in response to lipid antigen variants.^40^ Due to differences in antigen presentation, Th1 lipid antigens are predominantly presented by CD8α^+^ DCs that produce a large amount of IL12 and subsequently promote Th1 responses in vivo, whereas Th2 lipid antigens are efficiently presented by multiple CD1d-expressing cell types, most of which are not IL12-producing cells.^22^ However, it is paradoxical that CD8α^+^ DCs also present Th2 lipid antigens but fail to produce IL12 (Fig. 1b, f),^41^ and that difference is responsible for the minor IFNγ production in response to Th2 lipid antigens. It is well known that iNKT cells promote IL12 production in DCs through CD40L-CD40 interactions.^42,43^ However, similar amounts of CD40L and CD40 were expressed by iNKT cells and DCs, respectively, in response to distinct lipid antigen variants (Fig. 1d, Supplementary Fig. S6). Although lipid antigen variants have been shown to modulate the expression of costimulatory and coinhibitory molecules in DCs, these molecules are not involved in regulating the production of IL12.^41^ The mechanisms underlying the differences in IL12 production in response to different lipid antigen variants remain unclear. Our results demonstrate a new way to regulate IL12 production in DCs by modulating cytokine secretory routes. Compared to multidirectional secretion, polarized secretion concentrates cytokines at the cleft between an iNKT cell and APC, which benefits cytokine-mediated cell crosstalk. IL4 promoted IL12 production in DCs in a dose-dependent manner (Supplementary Fig. S1B); therefore, the accumulation of IL4 at the iNKT cell-DC IS as a result of polarized secretion favors the activation of the IL4R-STAT6 pathway and the production of IL12 in DCs. In contrast, although Th2 lipid antigen variants were able to induce the production of the same level or even more IL4 than was αGC, the nonpolarized manner of secretion failed to promote IL12 production efficiently and thereby caused Th2-biased cytokine responses. These results demonstrate that cytokines regulate immune responses not only through their amounts but also through their spatial distributions. IL4 is a well-known Th2-promoting cytokine.^44,45^ Paradoxically, IL4 from iNKT cells promoted STAT6 phosphorylation and IL12 production in DCs and enhanced iNKT cell-mediated Th1 responses (Fig. 2). Another study reported that TLR ligand-activated DCs but not helminth Ag SEA-activated DCs increase their IL12 production in response to IL4.^25^ It is possible that the influence of IL4 on DCs is dependent on the activation signaling pathway.

Previous studies indicate different secretory routes for IL4 and IFNγ in Th2 and Th1 CD4^+^ T cells, respectively.^1^ It is unclear whether iNKT cells release IL4 and IFNγ through different routes. IFNγ production exhibited kinetics different from those of IL4 production, and only a small amount of IFNγ was detected after activating iNKT cells for 4 h. In the small number of IFNγ-positive iNKT cells, the Th1 lipid antigen-induced polarized secretion, whereas the Th2 lipid antigen caused multidirectional secretion (Supplementary Fig. S7A). Interestingly, the release sites of IL4 and IFNγ overlapped. Moreover, intracellular IL4 and IFNγ were completely colocalized at the IS in iNKT cells activated by αGC (Supplementary Fig. S7B). In agreement with the above results, when nocodazole was used to disturb the polarization of the MTOC, intracellular IL4 vesicles and IFNγ vesicles were dispersed around the nucleus and colocalized as well (Supplementary Fig. S7B). Therefore, IL4 and IFNγ are released from iNKT cells in the same vesicles.

Maintaining the polarization of IL4 by taxol treatment significantly enhanced αGC acC8-induced STAT6 phosphorylation (Fig. 5d) and IL12 production (Fig. 5e) in DCs and IFNγ production (Fig. 5f) in iNKT cells. The spatial distributions of cytokines influence cell functions more significantly in vivo than in vitro. Quick diffusion of nonpolarized cytokines is expected in vivo due to extracellular fluid flow, resulting in a low local concentration at the IS. However, in an in vitro system, the accumulation of cytokines in the culture medium would diminish differences in cytokine concentration at the IS caused by distinct secretory routes. These possibilities explain the previous findings that Th2 lipid antigens induce undetectable IL12 production in vivo, but cause a large amount of IL12 production in DCs in vitro after long-term cell culture.^22^ When taxol was used in our studies to stabilize the MTOC and maintain the polarized secretion of IL4 in response to αGC acC8, increased IL12p70 production in DCs was observed only shortly after activation when the concentration of IL4 in the supernatant was low. Moreover, Cdc42 has been shown to regulate the formation of the IS in several studies.^34,35^ It is possible that lipid antigen variants differ in their ability to maintain IS formation. Again, the IS is less stable in vivo than in vitro due to the high motility of immune cells and fluid shear stress in vivo. It is rational that an unsffig IS induced by Th2 lipid antigens further inhibits the polarization and accumulation of IL4 at the IS, hinders the activation of the IL4R-STAT6 pathway in DCs, and contributes to Th2-biased cytokine responses.

The distinct secretory routes in iNKT cells were controlled by the type of lipid antigen by modulating the protein level of Cdc42 (Fig. 6c, d). Cdc42 but not DGK maintained the reorientation of the MTOC toward the IS in iNKT cells (Supplementary Fig. S5 and Fig. 6a). Th2 lipid antigens induced the degradation of Cdc42 by the proteasome (Fig. 6e). However, the underlying mechanisms are still unclear. Previous studies suggest that lipid antigen variants are distinctly related to lipid raft microdomains in APCs,^21,46^ which might contribute to the different immune responses.^46^ Reduced Cdc42 expression was detected in tumor-infiltrating iNKT cells and was associated with impaired IL4 polarization. In agreement with the observed multidirectional IL4 secretion, diminished STAT6 activation and dampened IFNγ production were detected in vivo in intratumoral DCs and iNKT cells, respectively (Fig. 7). These results demonstrated an abnormal secretory route for IL4 and impaired iNKT cell-DC crosstalk in tumors. iNKT cell-mediated Th1 responses play important roles in antitumor immunity.^37^ Therefore, the suppression of iNKT cell-mediated Th1 responses would favor tumor growth. The factors causing the reduction in Cdc42 expression in the tumor microenvironment remain unclear, and whether endogenous lipid antigens are involved and contribute to the progression of tumors requires further investigation.

Overall, our results demonstrate that polarized secretion rather than multidirectional secretion of IL4 in iNKT cells promotes crosstalk between iNKT cells and DCs and enhances iNKT cell-mediated Th1 responses (Supplementary Fig. S8). In addition to the amount of a cytokine, the spatial distribution of the cytokine could be another important factor modulating immune responses in vivo.

## Methods

### Mice

C57BL/6 WT mice were purchased from Beijing Vital River Laboratory Animal Technology (Beijing, China). *Vα14* Tg. *Cxcr6*^*gfp/+*^ mice on the C57BL/6 background were provided by Dr. Albert Bendelac (The University of Chicago).^22^ All mice were bred in a specific pathogen-free facility at the University of Science and Technology of China. All animal experiments were approved by the Institutional Animal Care and Use Committee of the University of Science and Technology of China.

Indicated lipid antigens (2 μg per mouse) were intraperitoneally injected into WT, *Il4ra*^*−/−*^, or MC38 tumor-bearing mice. To measure DC activation and cytokine production in the serum, tissue, and serum samples were collected after 8 h. Cytokines produced by intratumoral iNKT cells were measured with cytokine secretion Assay-Detection Kit (Miltenyi Biotec).

### Reagents and inhibitors

PBS57, αGC acC8, and αGC acC20:2 were provided by Dr. Albert Bendelac (The University of Chicago) and Dr. Paul B. Savage (Brigham Young University). αGC was purchased from Avanti Polar Lipids, and OCH was purchased from Cayman Chemical. A CD1d-PBS57 tetramer was provided by the NIH Tetramer Core Facility. DGK II, ZCL278, nocodazole, taxol, and MG132 were purchased from Sigma-Aldrich or MedChemExpress.

### Cell culture and a cytokine secretion assay

RBL.CD1d cells or splenic DCs were seeded on poly-l-lysine-coated coverslips and pulsed with 1 μg/ml αGC, PBS57, αGC acC8, αGC acC20:2, or OCH overnight. Then, the cells were cocultured with splenic iNKT cells isolated from *Vα14* Tg mice with anti-CD4 microbeads (Miltenyi Biotec). To overexpress GFP-Cdc42, GFP-Cdc42V12, or GFP-Cdc42N17, iNKT cells were transfected using the Amaxa^®^ Mouse T Cell Nucleofector^®^ Kit. To study the influences of inhibitors on IL4 secretion, DGK II (50 μM) was added a half hour before or 2 h after activation as indicated. ZCL278 (100 μM) and nocodazole (33 μM) were added into the culture medium during the last 30 min, taxol (100 nM) was added 2 h after activation, and MG132 (5 μM) was added at the beginning of activation. To detect the secretory sites of IL4 in iNKT cells, the IL4 secretion Assay-Detection Kit (Miltenyi Biotec) was used. iNKT cells cocultured with APCs for the indicated time points were labeled with a mouse IL4 capture reagent on ice for 5 min, transferred to a warm medium, and incubated at 37 °C for 45 min. After washing, the cells were stained with a phycoerythrin (PE)-conjugated anti-mouse IL4 detection antibody for 10 min on ice.

### MTOC polarization assay

iNKT cells activated by antigen-loaded RBL.CD1d cells or antigen-loaded splenic DCs were fixed for 15 min with 4% paraformaldehyde. To observe the MTOC, the cells were permeabilized with 0.2% Triton X-100 (Sigma Aldrich), blocked with 5% bovine serum albumin (BSA), and then stained with an anti-α-tubulin Alexa Fluor 488-conjugated antibody (Invitrogen) at room temperature for 1 h. For quantification of MTOC polarization, line “a” paralleling the interface between an APC and iNKT cell was drawn at the IS. Line “b” paralleling line “a” was drawn through the MTOC. Then, line “c”, which indicated the vertical distance between line “a” and line “b”, was used to quantify MTOC polarity. Images were acquired with an LSM 710 confocal microscope (Zeiss) with a ×100 objective. Data were analyzed with ImageJ software.

### Activation of freshly isolated DCs

DCs were enriched from the spleen of C57BL/6 WT mice with an anti-CD11c PE-conjugated monoclonal antibody and anti-PE microbeads (Miltenyi Biotec). Then, CD11c^hi^ B220^−^ DCs were sorted by a BD FACS Aria II. GFP^hi^ iNKT cells were sorted from the liver of *Vα14* Tg. *Cxcr6*^*gfp/+*^ mice by a BD FACS Aria II. Sorted DCs and iNKT cells were cocultured in 96-well round-bottomed plates in the presence of 1 μg/ml αGC or αGC acC8. LEAF-purified immunoglobulin G (IgG) 2b (5 μg/ml), anti-IL4 (5 μg/ml), anti-IL4R (5 μg/ml), and anti-IL12 (5 μg/ml) antibodies were added into the coculture system. After 8 h, IL12p70, IFNγ, and IL4 levels in the supernatants were measured by a cytometric bead array (BD Biosciences), and the phosphorylation of STAT6 in DCs was measured by flow cytometry. To investigate the influences of IL4 on DCs, freshly isolated DCs (2 × 10^5^) were stimulated with 1 μg/ml LPS (Sigma-Aldrich) plus murine recombinant IL4 (mrIL4) (PeproTech) at the indicated concentrations. All antibodies were purchased from BioLegend or BD Biosciences.

### BMDC culture and transfer

BMDCs were generated as previously described.^25^ In brief, total BM cells from WT or *Il4ra*^*−/−*^ mice were cultured for 6 days in RPMI medium supplemented with 10% fetal bovine serum and mr granulocyte-macrophage colony stimulating factor (10 ng/ml, PeproTech). Then, the BMDCs were harvested and incubated with or without αGC (1 μg/ml) overnight. After labeling with CFSE (0.2 μM, Invitrogen), the BMDCs (1 × 10^6^ cells per mouse) were injected intravenously into WT recipient mice. Tissue and serum samples were collected after 8 h.

### Flow cytometry

Cell surface staining was performed as previously reported. Briefly, cells were blocked with purified anti-CD16/32 and then stained with fluorochrome-conjugated monoclonal antibodies against CD11c (N418), CD11b (M1/70), CD80 (16-10A1), CD86 (24F), CD40 (HM40-3), B220 (RA3-6B2), and TCRβ (H57-597) and a CD1d-PBS57 tetramer. For pSTAT6 staining, cells were fixed immediately with prewarmed 2% paraformaldehyde and then permeabilized with ice-cold 90% methanol. Antibodies against pSTAT6 (CHI2S4N) and CD11c (N418) were incubated with the cells for 1 h on ice. All antibodies were purchased from eBioscience or BioLegend. The cells were acquired on a FACSVerse flow cytometer (BD Biosciences), and data were analyzed with FlowJo software (TreeStar).

### Immunofluorescence microscopy

*Vα14* Tg. *Cxcr6*^*gfp/+*^ mice were intraperitoneally immunized with αGC or αGC acC20:2 (2 μg per mouse). Two hours after injection, spleens were harvested, fixed with 4% paraformaldehyde for 2 h, and then dehydrated in 30% sucrose overnight before embedding in OCT medium. Frozen sections (20-μm thick) were blocked with purified anti-CD16/32 in a 5% BSA-PBS (phosphate-buffered saline) buffer and then stained with antibodies at 4 °C overnight. Images were taken with an LSM 710 confocal microscope (Zeiss) with a ×10 objective. Data were analyzed with the ImageJ software.

### Western blotting

RBL.CD1d cells were plated in a six-well plate (1 × 10^6^ cells per well) in the presence of the indicated lipid antigen (1 μg/ml) overnight and then cocultured with hepatic lymphocytes from *Vα14* Tg. *Cxcr6*^*gfp/+*^ mice for 4 h. GFP-positive iNKT cells were sorted sequentially and used for western blotting. The antibodies used were as follows: rabbit anti-Cdc42 (Abcam), mouse anti-Cdc42-GTP (New East), and horseradish peroxidase-conjugated anti-rabbit IgG (Proteintech). To detect GTP-bound Cdc42, iNKT cells were lysed in an NP-40 lysis buffer, and Cdc42-GTP was pulled down by an anti-Cdc42-GTP antibody. Then, GTP-bound Cdc42 was detected by an anti-Cdc42 antibody.

### Statistical analysis

Statistical analysis was performed using GraphPad Prism 5. An unpaired Student’s *t* test, the Mann–Whitney *U* test, one-way analysis of variance (ANOVA) with the Tukey’s comparison post test or two-way ANOVA with the Bonferroni correction post test was used to determine significant differences. For all experiments, significance was defined as a *P* value < 0.05.

## Supplementary information

supplemental material

## Data Availability

All data are available within the article (as figure source data or Supplementary Information Files) and/or from the authors on request.
