# Supplementary material for: Spatial distribution of IL4 controls iNKT cell-DC crosstalk in tumors
Source: Cell Mol Immunol. 2019 Jun 3;17(5):496–506. doi: 10.1038/s41423-019-0243-z (PMC7192838; doi:10.1038/s41423-019-0243-z)
Supplement: Supplementary file 1 — supplemental material [file 41423_2019_243_MOESM1_ESM.docx]

**Supplementary files**


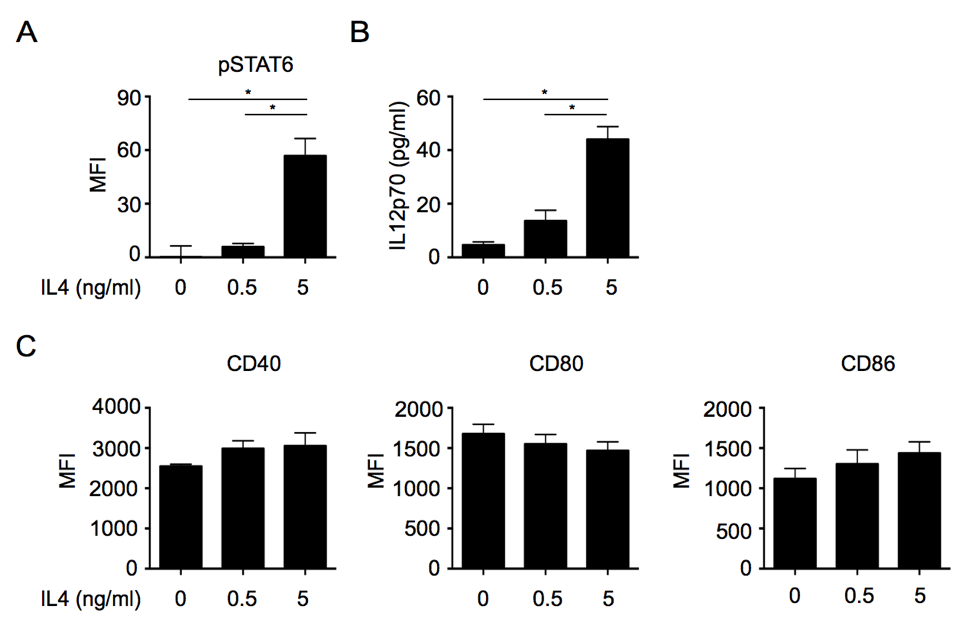


**Figure S1. IL4 significantly increases phosphorylation of STAT6 and IL12 production in DCs activated by LPS.**

(**A-C**) Influences of IL4 on phosphorylation of STAT6 (**A**), IL12 production (**B**) and CD40, CD80, CD86 expression (**C**) in LPS-activated DCs at indicated concentrations. Data are mean ± SEM of four independent experiments. Statistical analysis was performed using one-way ANOVA with a Tukey comparison. *P < 0.05.

**
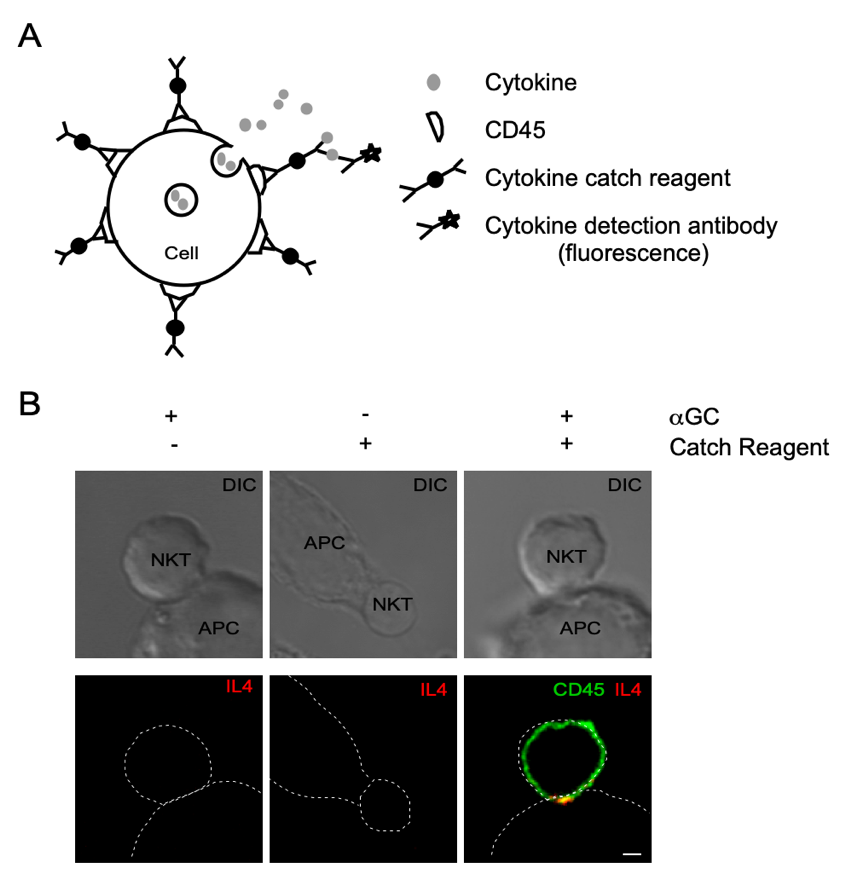
**

**Figure S2.** **Strategy to detect IL4 secretory sites.**

(**A**) Strategy to detect IL4 secretory sites. (**B**) Validation of detecting method as in (**A**). Dotted lines indicate cell boundaries. Scale bars, 2 μm. Data are representative of three independent experiments.

**
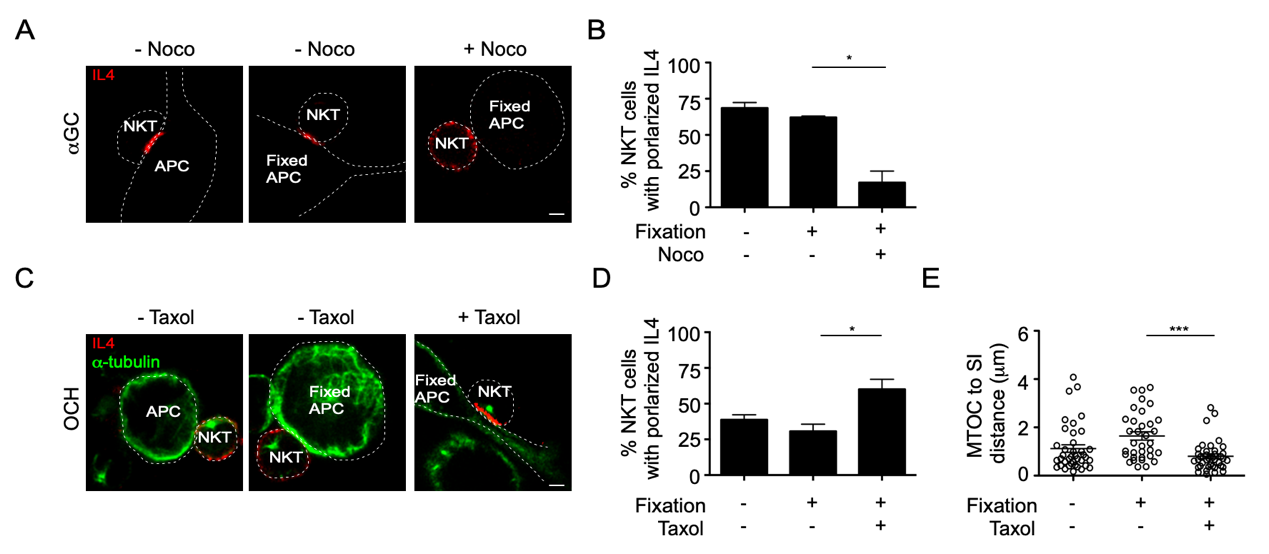
**

**Figure S3.** **Influences of Nocodazole and Taxol on iNKT cells activated by pre-fixed and antigen-pulsed RBL.CD1d cells.**

(**A**, **B**) Influences of Nocodazole (33 μM) on IL4 secretory sites in iNKT cells activated by pre-fixed αGC-pulsed RBL.CD1d cells for 4 hours. (**C**-**E**) Influences of Taxol (100 nM) on distance from MTOC to SI (**C**, **E**) and on IL4 polarization (**C**, **D**) in iNKT cells activated by pre-fixed OCH-pulsed RBL.CD1d cells for 4 hours. Dotted lines indicate cell boundaries. Scale bars, 2 μm. Data are representative of (**A**, **C**) or are mean ± SEM of three independent experiments (**B**, **D**), or are mean ± SEM of more than 60 cells per group (**E**). Statistical analysis was performed using Student’s *t*-test. *P < 0.05; ***P < 0.001.

**
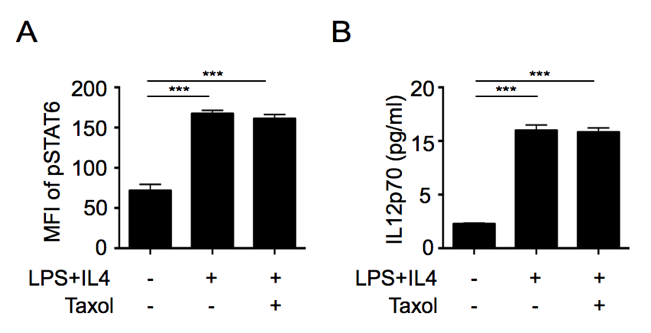
**

**Figure S4.** **Taxol shows no direct effect on DCs**

(**A**, **B**) Influences of Taxol on phosphorylation of STAT6 (**A**) and on IL12 production (**B**) in LPS-activated DCs. Data are mean ± SEM of three independent experiments. Statistical analysis was performed using one-way ANOVA with a Tukey comparison. ***P < 0.001.

**
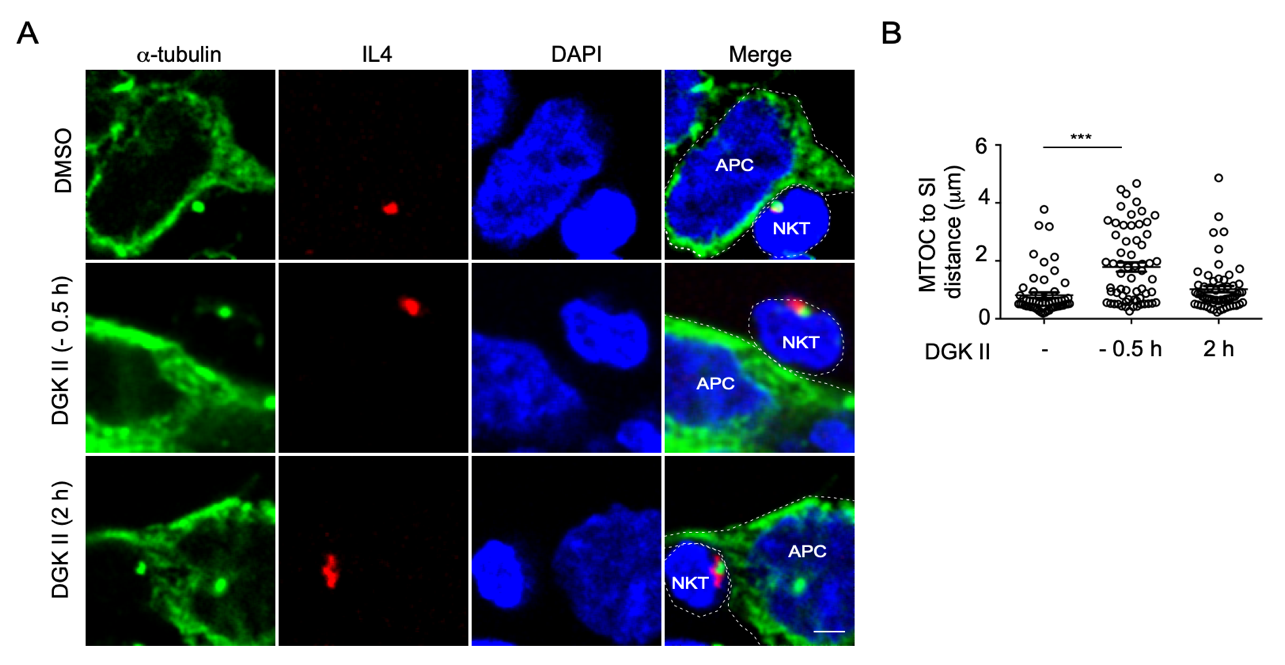
**

**Fig S5. DGK controls initiation of MTOC polarization.**

(**A**, **B**) Influences of DGKII (50 μM) on MTOC polarization, intracellular IL4 polarization (**A**), and on distance from MTOC to SI (**B**) in iNKT cells activated by αGC-pulsed RBL.CD1d cells for 4 hours. DGKII was added to culture medium at indicated time points. Dotted lines indicate cell boundaries. Scale bars, 2 μm. Data are representative of three independent experiments (**A**) or are mean ± SEM of more than 55 cells per group (**B**). Statistical analysis was performed using Student’s *t*-test. ***P < 0.001.


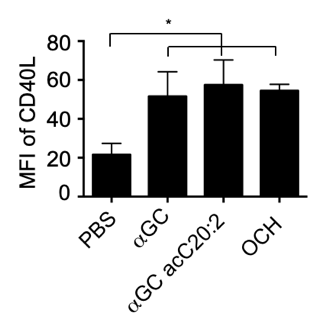


**Figure S6. Lipid antigen variants induce similar expression of CD40L on iNKT cell surface.**

Expression of CD40L in splenic iNKT cells from WT mice receiving indicated lipid antigens. Data are mean ± SEM of nine mice per group. Statistical analysis was performed using a one-way ANOVA with a Tukey comparison. *P < 0.05.


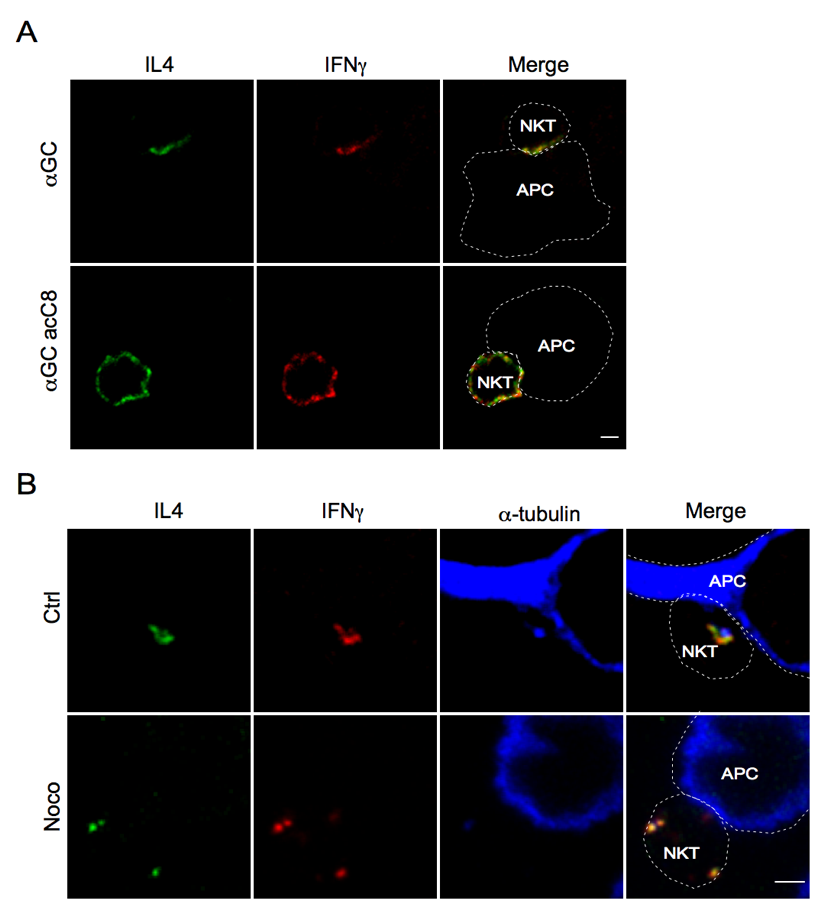


**Fig S7. Spatial distribution of IL4 and IFNγ in iNKT cells.**

(**A**) Secretory sites of IL4 (green) and IFNγ (red) in iNKT cells activated by αGC-pulsed RBL.CD1d cells. (**B**) Co-localization of intracellular IL4 (green) and IFNγ (red) in iNKT cells activated by αGC-pulsed RBL.CD1d cells in the presence and absence of Nocodazole (33 μM). Blue, tubulin. Dotted lines indicate cell boundaries. Scale bars, 2 μm. Data are representative of three independent experiments.

**
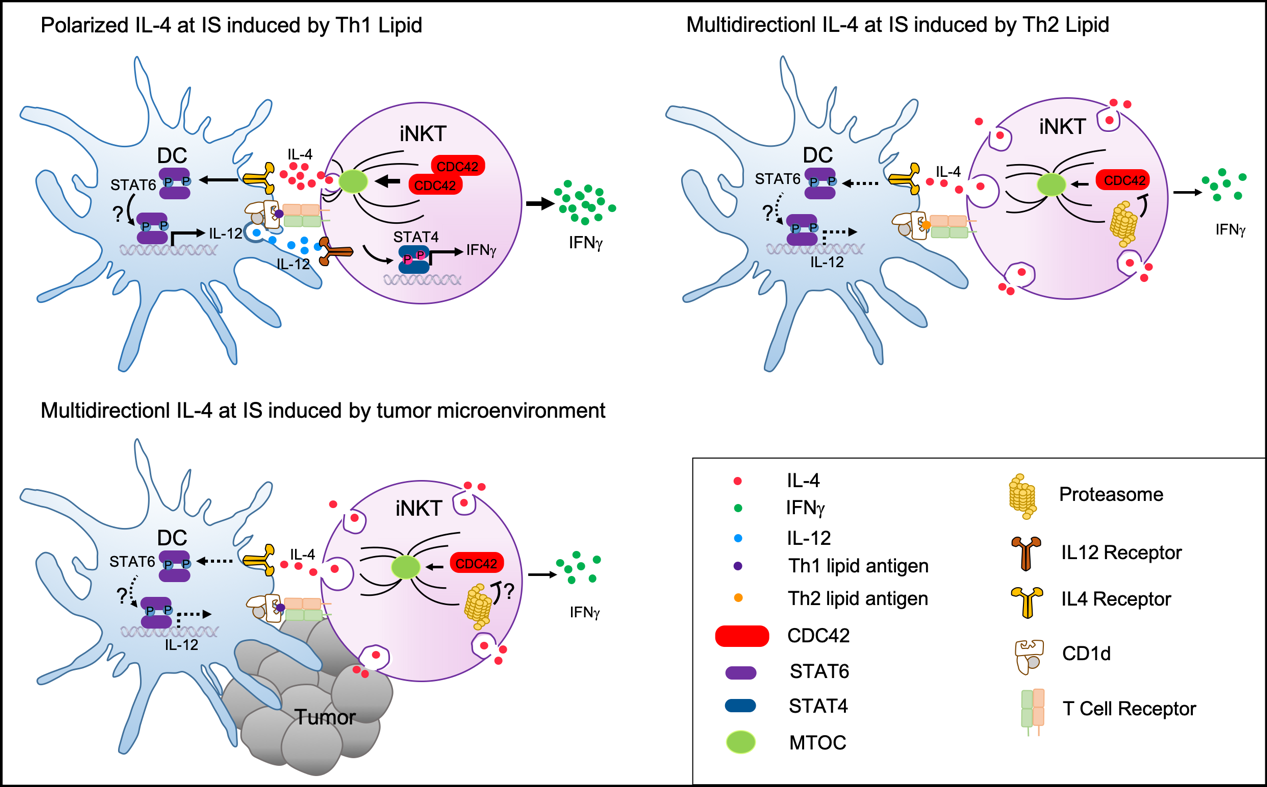
**

**Figure S8. Working model.**

Polarization of IL4 at immunological synapse promotes iNKT-DC crosstalk and iNKT cell-mediated Th1 responses. αGC activated iNKT cells and induced polarization of IL4 toward immunological synapse in normal condition. Reduced Cdc42 in response to Th2 lipid antigens or caused by tumor microenvironment failed to maintain MTOC polarization and hence caused multidirectional secretion of IL4 in iNKT cells. Polarized secretion rather than multidirectional secretion of IL4 enhanced IL4R-STAT6 activation and IL12 production in DCs, and consequently promoted IFNγ production in iNKT cells.
